# Beyond chemical composition: solvent pretreatment modulates supramolecular assembly and interactions with MRSA to enhance antibacterial efficacy of *Scutellariae Radix*-*Coptidis Rhizoma*

**DOI:** 10.1186/s13020-026-01460-7

**Published:** 2026-07-17

**Authors:** Yiqing Huang, Yihang Zhao, Jichang Wei, Jihui Lu, Zhiwei Wang, Gen Li, Helong Xu, Yuanhua Li, Xuemei Huang, Penglong Wang

**Affiliations:** School of Chinese Pharmacy, Beijing University of Chinese Medicine, Sunshine South Street, Fangshan District , Beijing, 102488 China

**Keywords:** *Scutellariae Radix*, *Coptidis Rhizoma*, Solvent pretreatment, Supramolecular assembly, MRSA

## Abstract

**Background:**

*Scutellariae Radix*-*Coptidis Rhizoma* herb pair is a classic traditional Chinese medicine (TCM) combination with heat-clearing, dampness-drying and detoxifying effects, extensively applied clinically against bacterial infections. Traditional water decoction and ethanol pretreatment are mainstream extraction approaches, whereas existing comparisons merely emphasize quantitative variations of bioactive compounds, neglecting the underlying supramolecular assembly differences of effective substances. Comparative analysis of solvent-induced supramolecular variations provides a novel perspective to interpret differential pharmacodynamic material bases of TCM formulas.

**Methods:**

Macroscopic and microscopic morphologies of HH-0 and HH-50 were firstly characterized by visual inspection, SEM and DLS. Their phytochemical profiles were subsequently analyzed by UHPLC-Q-Orbitrap HRMS and HPLC. Antibacterial activities against MRSA were further quantified via turbidimetry and plate assays. UV, FT-IR and MD simulations revealed divergent supramolecular assembly behaviors. Finally, untargeted metabolomics and assembly-peptidoglycan interaction simulations elucidated the underlying anti-MRSA mechanisms.

**Results:**

The solvent pretreatment endowed HH-0 and HH-50 supramolecules with distinct characteristics. HH-50 featured rougher supramolecular surfaces and higher Zeta potential with favorable dispersibility. The two supramolecules shared consistent active constituents, and HH-50 showed elevated berberine abundance and enhanced anti-MRSA activity. Notably, HH-50 maintained superior antibacterial efficacy and interference against peptidoglycan synthesis and energy metabolism even at identical berberine doses. This confirmed that divergent supramolecular conformations determined antibacterial differences, as diminished hydrogen bonds and weakened van der Waals and electrostatic interactions enhanced the binding affinity between assemblies and MRSA.

**Conclusion:**

Beyond chemical composition, solvent pretreatment (water versus 50% ethanol) markedly modulates supramolecular assembly behaviors and subsequent assembly-bacteria interactions, thereby regulating the anti-MRSA efficacy of the *Scutellariae Radix*-*Coptidis Rhizoma* herb pair. This work emphasizes that supramolecular configurations, rather than simple differences in chemical contents, dominate the pharmacological activities of traditional Chinese medicine preparations.

**Graphical Abstract:**

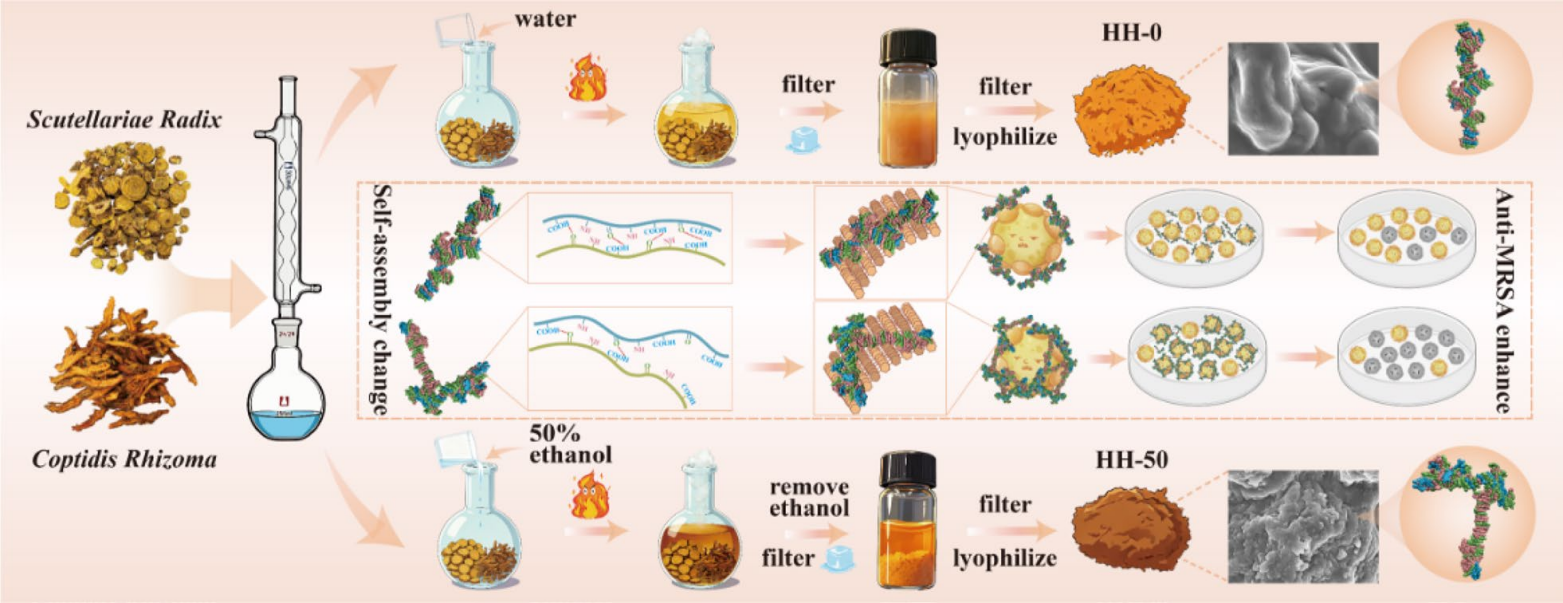

**Supplementary Information:**

The online version contains supplementary material available at 10.1186/s13020-026-01460-7.

## Introduction

In recent years, the spontaneous self-assembly of active components from traditional Chinese medicine (TCM) into supramolecular assemblies driven by noncovalent interactions including hydrogen bonds, π-π stacking and other intermolecular weak forces have emerged as a hot research direction in the modernization of TCM and gained widespread attention from global scholars [1–3]. Such multicomponent supramolecular systems can mimic the “multi-component, multi-target, and synergistic” characteristics of TCM formulas, which is highly consistent with the holistic view of TCM, breaking through the limitations of the traditional single-component-oriented research paradigm for pharmacodynamic substances, and providing a novel research perspective and technical approach for elucidating the molecular mechanisms of multicomponent synergy and revealing the scientific connotation of TCM formulas [4]. A large number of studies have shown that the formation of TCM supramolecules is not a random process but a complex and dynamic process coordinately regulated by multiple factors, among which solvent [5–7], temperature [8–10], pH value [11, 12], and the ratio of active components [13, 14] can exert significant regulatory effects on supramolecular assembly by changing the strength and type of noncovalent interactions between components.

*Scutellariae Radix* (HQ) and *Coptidis Rhizoma* (HL) are a classic herb pair with the therapeutic effects of “clearing heat, drying dampness, purging fire and detoxifying”, originating from renowned classical formulas such as Gegen Qinlian Decoction, Huanglian Jiedu Decoction and Banxia Xiexin Decoction [15–18]. It exhibits broad-spectrum antibacterial activity in modern clinical and pharmacological studies, especially showing significant inhibitory effects against Gram-positive bacteria including methicillin-resistant *Staphylococcus aureus* (MRSA), *Staphylococcus epidermidis* and streptococci, and investigations into its pharmacodynamic material basis and mechanism have attracted extensive attention [19, 20]. Our previous studies have confirmed that the supramolecular system formed by HQ-HL during water decoction serves as an important pharmacodynamic material basis for its antibacterial activity [21], and have illustrated that temperature exerts a remarkable influence on supramolecular morphology during its formation, further affecting its antibacterial efficacy [10]. In addition to traditional decoctions using water as the sole solvent, the HQ-HL herb pair has been developed into various Chinese patent medicinal preparations including tablets, capsules and granules to meet the requirements of modern clinical applications [15]. Compared with traditional decoctions, a key difference lies in the introduction of ethanol during pretreatment to optimize extraction efficiency [22, 23]. Of particular note, except for special dosage forms such as tinctures and medicinal liquors, ethanol is only employed in the pretreatment stage during the preparation of modern herbal medicines, and is typically completely removed through subsequent concentration, drying and other technological links, enabling the final pharmaceutical system to revert to an aqueous system [24]. To date, most existing comparative researches on aqueous and ethanolic processing of this herb pair merely focused on discrepancies in the category and content of flavonoid and alkaloid components. For instance, abundant data verified that water extraction tends to retain more polar flavonoid components, while extraction using ethanol as solvent elevates the yields of lipophilic aglycones and free alkaloid [25–27]. Nevertheless, few reports have systematically illuminated how extraction solvent modulates the supramolecular self-assembly process of HQ and HL, as well as the subsequent structure–activity relationship against MRSA [28, 29]. Given that solvent environments dominantly govern supramolecular assembly, different solvents regulate component solubility and intermolecular interactions via differences in polarity, hydrogen-bonding ability and solvation properties, thereby affecting the formation, aggregation state, morphological characteristics and assembly patterns of supramolecules [30–32]. More importantly, supramolecular assembly structures are tightly associated with biological functions, as their morphology and aggregation state directly influence solubility, stability, bioavailability and target binding capacity, thus determining the final antibacterial efficacy [33–35]. Accordingly, an important scientific uncertainty remains to be clarified: whether the transient ethanol environment during the pretreatment can modulate the supramolecular assembly process, thereby leading to irreversible changes in the supramolecular assembly even after the final reversion to the aqueous system, and further affecting its biological activity.

Against this background, in this study, the classic herb pair HQ-HL was used as the research object to systematically compare the differences in supramolecular assembly, antibacterial activity and interactions with MRSA between two aqueous systems: the direct water-extracted system (HH-0), and the 50% ethanol-pretreated system that undergoes full ethanol volatilization to reconstruct aqueous phase (HH-50). Notably, 50% ethanol was selected in accordance with the Chinese Pharmacopoeia-specified extraction conditions for classical Gegen Qinlian pills, which enables balanced enrichment of both polar flavonoids and lipophilic alkaloids and represents a commonly adopted processing solvent for modern Chinese medicinal preparations [24]. We further explored the regulatory effect of the transient ethanol environment during pretreatment on supramolecular configurations and their antibacterial activity under unified aqueous reconstruction conditions. The results showed that the mac- and microscopic morphology and assembly modes of the two HQ-HL supramolecular systems in aqueous medium were significantly distinct. Such discrepancies further alter the supramolecule-bacterium interactions, thereby resulting in divergent anti-MRSA activities. These findings indicate that the therapeutic efficacy of classical Chinese herbal pairs can be precisely optimized via supramolecular configuration regulation, transcending mere quantitative differences in chemical constituents.

## Materials and methods

### Materials

*Scutellariae Radix* (HQ, Batch No. 250208006,*Scutellaria baicalensis* Georgi., dried root) originating from Chifeng, Inner Mongolia and *Coptidis Rhizoma* (HL, Batch No. 250218001, *Coptis chinensis* Franch., dried rhizome) originating from Yaan, Sichuan Province were purchased from Tongrentang (Beijing, China). Ethanol, 2.5% glutaraldehyde solution, methanol, acetone was purchased from Beijing Chemical Plant (Beijing, China). Phosphate (PBS) buffer was purchased from biodee. (Beijing, China). Vitamin K was purchased from Aladdin Reagent Co., Ltd. (Shanghai, China). Methicillin-resistant *Staphylococcus aureus* (MRSA, ATCC 43300) was acquired from school of Life Sciences, Beijing University of Chinese Medicine (Beijing, China); nutrient broth (Batch No. 20240704) and nutrient agar (Batch No. 20240530) were purchased from Solarbio biotechnology Co., Ltd (Beijing, China).

### Sample preparation

6 g of HQ and HL were weighed separately at a 1:1 mass ratio, and subjected to parallel extraction with either deionized water or 50% aqueous ethanol. Briefly, herbal slices were soaked in tenfold volume of solvent for 30 min prior to 1 h reflux extraction under sustained boiling. The reflux temperature was maintained at 100 °C for water and at 87 ± 2 °C for 50% aqueous ethanol, respectively. After hot filtration to remove residues, the aqueous decoction was directly centrifuged, whereas the ethanolic decoction was rotary-evaporated to remove ethanol prior to centrifugation at 8000 r·min⁻^1^ for 30 min. The supernatant and supramolecular fractions were collected respectively and freeze-dried to obtain lyophilized powder HH-0 (water-extracted supramolecules) and lyophilized powder HH-50 (50% ethanol-pretreated supramolecules), and the extract yield was calculated. All experiments were performed in triplicate.

### Dynamic light scattering characterization (DLS)

HH-0 and HH-50 were individually dispersed in deionized water at a same concentration and measured the Zeta potential of each sample by Zetasizer Nano ZS 90 (Maleven, UK). The results were repeated three times and averaged.

### Scanning electron microscopy characterization (SEM)

The samples were dispersed in deionized water to the same concentration and 2 μL was transferred to silicon wafers for drying naturally at room temperature. After spraying gold on the surface (working voltage was 10.0 kV), the morphology and particle size of each sample was observed by SEM (Hitachi SU-8020, Hitachi, Japan).

### In vitro antibacterial experiment of HH-0 and HH-50 at identical mass dosages

MRSA (ATCC 43300) was inoculated into nutrient broth for resuscitation, followed by streaking onto nutrient agar plates using an inoculating loop. The plates were incubated at a constant temperature of 37 °C in a biochemical incubator. After colony formation, the passaging procedure was repeated to obtain the second-generation culture. A single colony was then picked and transferred into liquid medium for large-scale expansion. Subsequently, the MRSA suspension was diluted to a concentration of 2× 10^6^ CFU mL⁻^1^ and stored in a refrigerator until further use. All nutrient broth and agar medium applied in the entire experiment were prepared from a single batch.

Subsequently, the anti-MRSA activities of HH-0 and HH-50 were first compared by turbidimetric assay. Samples were diluted into drug suspensions in a 48-well plate with a final volume of 500 μL per well, and finally 30 μL of bacterial suspension was added to each well separately. The final concentration of drug suspension was 0.65, 0.55, 0.45, 0.35 and 0.25 mg·mL^−1^, and the concentrations of bacterial suspension were 2 × 10^6^ CFU·mL^−1^*.* Then, the plate was placed in a constant thermostatic incubator at 37 ℃ for 12 h. Subsequently, the bacterial survival rate was calculated by measuring the absorbance at 600 nm via microplate reader.

$${\text{Inhibition rate }}\left( \% \right)\, = \,{1}00 - \left( {{\mathrm{OD}}_{{{\mathrm{sample}}}} - {\mathrm{OD}}_{{{\mathrm{solvent}}}} } \right)/\left( {{\mathrm{OD}}_{{\text{blank bacteria}}} - {\mathrm{OD}}_{{{\mathrm{solvent}}}} } \right)\, \times \,{1}00\%$$

Moreover, the suspension in the 48-well plate at a 0.55 mg·mL⁻^1^ concentration was collected and diluted 1×10^4^ times. Then, 50 μL of the diluted solution was transferred onto a nutrient agar plate and spread evenly using a spreader. After incubation at 37 ℃ in a constant-temperature incubator for 18 h, the colony growth on the nutrient agar plate was observed.

### UHPLC-Q-orbitrap HRMS analysis

UHPLC-Q-Orbitrap HRMS analysis was performed using UltiMate 3000 liquid chromatographic system and coupled with a Q Exactive quadrupole-Orbitrap high-resolution mass spectrometry (Thermo Fisher Scientifc, Massachusetts, USA). Each sample was prepared at 1 mg·mL^−1^ and filtered through 0.22 μm microporous filter membrane. The determination was performed on a TC-C_18_ column (4.6 mm × 250 mm, 5 μm, Agilent). The column temperature was maintained at 30 °C. The mobile phase consisted of 0.1% formic acid water (A) and methanol (B). The gradient conditions were as follows:0–30 min, 4–98% B; The flow rate of mobile phase was 0.3 mL·min^−1^, and the injection volume was 5 μL. The ESI source was used to collect mass spectra in positive and negative ion mode in the m/z range of 150–1500. The conditions of ESI–MS were as follows: atomization pressure was 45 psi, nitrogen was dry gas, flow rate was 1.0 mL·min^−1^, and temperature was 350 °C. The capillary voltage was set at 3500 V.

### High performance liquid chromatography (HPLC) analysis

Qualitative and quantitative analysis of the samples was carried out by high-performance liquid chromatography (HPLC) (Waters e2695, USA) equipped with a 2988 PDA detector and a X-bridge C18 analytical column (4.6 mm × 250 mm, 5 μm). The column temperature was maintained at 25 °C, and the detection wavelength was set at 280 nm and 354 nm. The injection volume was 10 μL, and chromatographic peaks were identified by comparison of retention times and absorption spectra with reference standards. The mobile phase consisted of solvent A (aqueous 0.1% phosphoric acid) and solvent B (acetonitrile) with a gradient elution program at a flow rate of 1.0 mL·min⁻^1^. The gradient program was set as follows: 0–5 min, 5%-10% B; 5–10 min, 10%-15% B; 10–20 min, 15%-20% B; 20–30 min, 20%-23% B; 30–40 min, 23%-35% B; 40–42 min, 35%-5% B; 42–50 min, 5% B for re-equilibration of the column. Each sample was prepared at 0.5 mg·mL^−1^ and filtration through a 0.22 μm membrane filter before injection.

### In vitro antibacterial assay of HH-0 and HH-50 with identical berberine content

Utilizing the standardized second-generation MRSA inoculum (2 × 10^6^ CFU mL⁻^1^) and single-batch culture medium prepared above, the in vitro anti-MRSA activities of HH-0 and HH-50 with equivalent berberine content were determined via turbidimetric assay. Samples were serially diluted to various concentrations with nutrient broth in 48-well plates. Then, 30 μL of standardized MRSA inoculum (2 × 10^6^ CFU mL⁻^1^) was supplemented into each well, and the plates were incubated at 37 °C for 12 h. Then record the OD value of the suspension at 600 nm. Further cultured the bacterial suspension in the 48-well plate on the nutrient agar by using a flat plate.

### The bacterial staining and observation of bacterial morphology by SEM

After incubating MRSA in nutrient broth for 12 h, different groups of drugs were added and co-incubated for another 12 h. Then, collected bacteria by centrifugation (3000 rpm·min^−1^,10 min), and washed the collected bacteria with PBS for 3 times. One part of the bacteria was stained with green fluorescent precursor Calcein AM/PI, and the number of live and dead bacteria was observed under a laser confocal scanning microscope. The other part of the bacteria was fixed with 2.5% glutaraldehyde at 4 ℃ for 4 h, and washed with PBS. Then gradient dehydration with different concentrations of ethanol (50%, 80%, 95%, 100%) for 10 min each time, and the final sample of 2 μL was dropped on the single-side polished silicon wafer, and the water was naturally dried at room temperature. After gold spraying, the samples were placed in SEM with a working voltage of 15 kv for observation.

### UV–Visible absorption spectrometric determination (UV–Vis)

Each sample with the same concentration was prepared. The detection wavelength of the UV–visible spectrophotometer was set at 200–600 nm and deionized water was used as a blank solution for full-wavelength scanning (UH5300, Hitachi, Japan).

### Fourier transform infrared spectroscopy determination (FT-IR)

The samples were put into a Fourier transform infrared spectrometer. Later, the infrared spectra of each sample were measured in the range of 400–4000 cm^−1^ with air as the background (ensor27, Bruker, USA).

### Non-targeted metabolomics analysis

Bacteria were collected following the methods for SEM sample preparation. The bacteria were resuspended in 1 mL of pre-cooled PBS (4 °C) and centrifuged twice (3000 rpm·min^−1^, 5 min, 4 °C) to remove residual nutrient broth and drug suspensions. Subsequently, 1 mL of PBS and 3 mL of methanol were added, followed by vortex mixing and ultrasonication at 15 °C for 1 h. The supernatant was collected after centrifugation (3000 rpm·min^−1^, 5 min, 4 °C), concentrated to dryness under nitrogen stream, and redissolved with a methanol-ultrapure water mixture (1:3, v/v). The solution was filtered through a 0.22 μm microporous filter membrane for analysis.

Data processing and statistical analysis: Raw data were preprocessed using Progenesis QI software (Waters MS Technologies, Manchester, UK) for peak alignment, retention time correction, and feature extraction. MetaboAnalyst 6.0 was employed for data filtering, missing value imputation, fold change (FC) calculation, and normalization. Multivariate statistical analyses, including principal component analysis (PCA) and partial least squares-discriminant analysis (PLS-DA), were performed using MetaboAnalyst 6.0. Potential differential metabolites were screened based on thresholds of P-value (< 0.05), variable importance in projection (VIP > 1), and fold change (FC > 1.5 or FC < 0.67). Pathway enrichment analysis of the identified metabolites was conducted via MetaboAnalyst 6.0 using the KEGG database.

### Molecular dynamics simulation (MD)

#### Molecular force field preparation

Geometry optimization and vibrational analysis of baicalin (BA), berberine (BBR), palmitic acid (PA), peptidoglycan (PG), and ethanol molecules were performed using the ORCA 5.0.3 software at the B97-3C level [36]. Single-point energy calculations were carried out at the PWPB95-D3/def2-QZVPP theoretical level [37–39] with the def2/J and def2-QZVPP/C auxiliary basis sets, accelerated by the RIJCOSX algorithm [40]. Wavefunction analysis was subsequently conducted using Multiwfn to derive RESP atomic charges. Topology files were then constructed based on the GAFF force field [41] using the Sobtop program.

#### Self-assembly molecular dynamics simulations

All molecular dynamics simulations were performed using GROMACS 2020.6. Initial systems containing 50 BA, 50 BBR, 20 PA molecules, and 3000 solvent molecules were constructed using Packmol. Energy minimization was conducted via the conjugate gradient method, followed by 200 ps of annealing simulation (0–100 ps, from 0 K to 373.15 K). Subsequently, 150 ns of NPT ensemble simulations were performed with staged temperature control: 0–40 ns at 373.15 K, 40–60 ns from 373.15 K to 298.15 K, and 60–150 ns at 298.15 K. For the HH-50 group, ethanol molecules were removed at 100 ns. Temperature and pressure were regulated using the V-rescale thermostat and Parrinello-Rahman barostat, respectively. All structural visualization was performed using VMD software [42].

### *Molecular dynamics simulations of bacterial interactions*

Representative final configurations obtained from the aforementioned self-assembly simulations were employed to construct periodic simulation boxes of 10 × 10 × 10 nm. A total of 500 N-acetylglucosamine molecules, used as a peptidoglycan mimic, were introduced into each system. Energy minimization, equilibration annealing, and 50 ns production molecular dynamics simulations were performed under identical protocols. Trajectory analysis was conducted to characterize the distinct interaction behaviors between HH-0, HH-50 and N-acetylglucosamine.

### Statistical analysis

Quantitative data were expressed as the means ± standard deviation of at least three independent assays. Significance analysis was evaluated by one-way analysis of variance, ANOVA. *P* < 0.05 was considered statistically significant.

## Results and discussion

### The morphology and characterization of HH-0 and HH-50

**Fig. 1:**
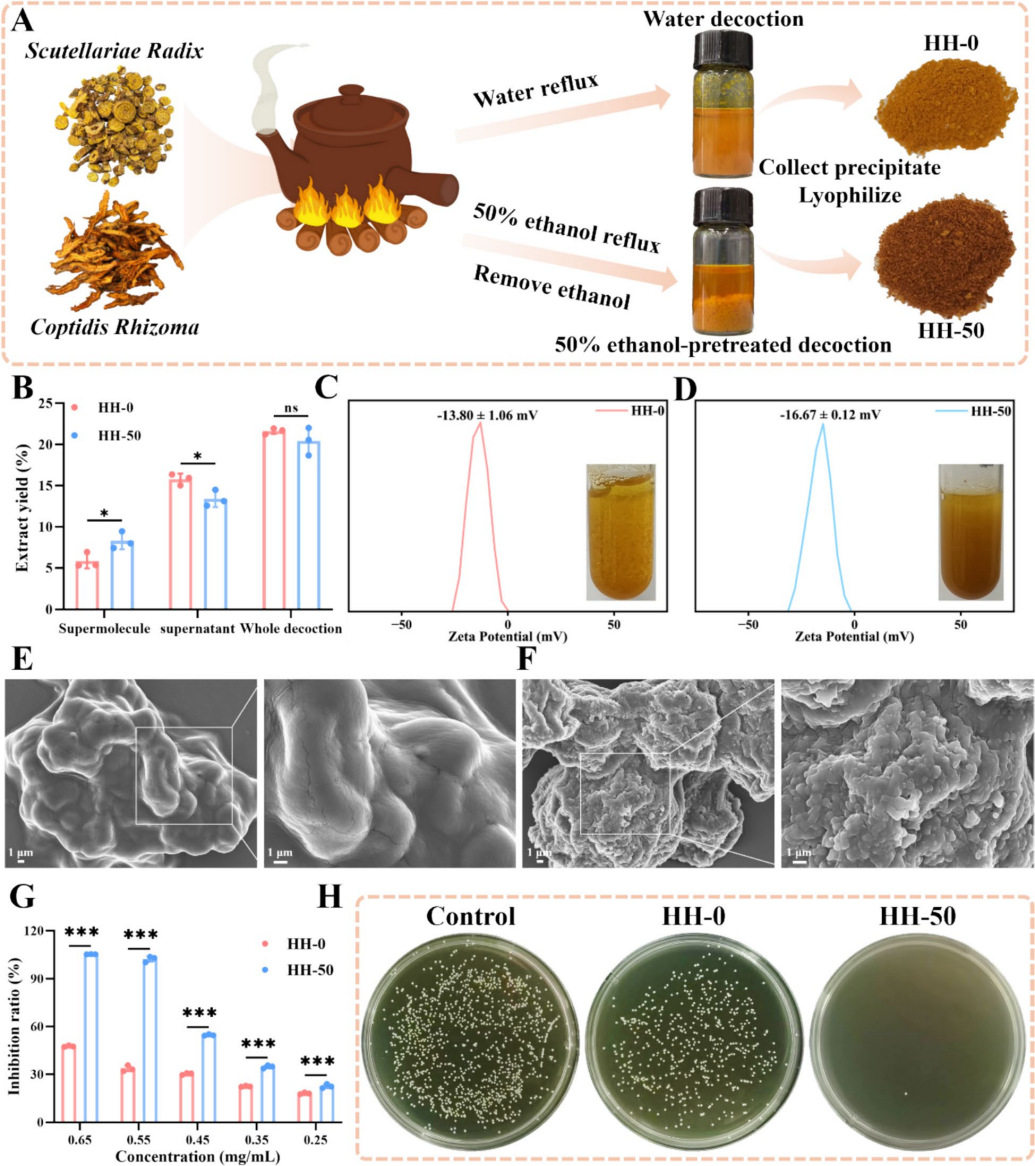
Characterization of supramolecular properties and antibacterial activities of HH-0 and HH-50. **A** The preparation process and macroscopic phase of HH-0 and HH-50. **B** The extract yield of HH-0 and HH-50. **C** Zeta potential of HH-0. **D** Zeta potential of HH-50. **E** SEM image of HH-0. **F** SEM image of HH-50. **G** The inhibition rate of HH-0 and HH-50 at different concentrations (n = 3). **H** Plate coating of each sample at 0.55 mg·mL⁻^1^

The macroscopic phase differences between the HQ-HL water co-decoction and 50% ethanol-pretreated decoction were distinct: the water co-decoction exhibited no obvious stratification but contained abundant fluffy and fine flocs; in contrast, the 50% ethanol-pretreated decoction showed clear stratification, with precipitates present as large flocculent aggregates. Moreover, the lyophilized powders of HH-0 and HH-50 displayed remarkable macroscopic disparities in color, with HH-50 possessing a finer texture than HH-0 (Fig. 1A). Statistical analysis of the extract yield demonstrated no significant difference in the overall extract yield between the HQ-HL water co-decoction and 50% ethanol-pretreated decoction (Fig. 1B). However, the supramolecular yield of HH-50 was markedly higher than that of HH-0 (*P* < 0.05), whereas the supernatant yield of the 50% ethanol-pretreated decoction was notably lower than that of the water co-decoction (*P* < 0.05). These findings indicate that 50% ethanol pretreatment promotes the supramolecular yield, suggesting that 50% ethanol pretreatment increases the probability of intermolecular interactions among dissolved components to form supramolecular assemblies. As show in Fig. 1C and D, combined with the DLS characterization results, it was found that HH-50 had higher absolute value of Zeta potential (16.67 mV) than HH-0 (13.80 mV), suggesting enhanced dispersion stability of supramolecular aggregates. Further observation via scanning electron microscopy (SEM) revealed that HH-0 appeared as aggregates with smooth surfaces (Fig. 1E), whereas HH-50 existed as agglomerates with granular protrusions on the surface (Fig. 1F), reflecting that HH-50 may possess a larger specific surface area than HH-0.

Numerous studies have demonstrated that the HQ-HL herb pair and its main components exert potent inhibitory activity against MRSA [22, 43]. Therefore, taking MRSA as a model pathogen, turbidimetry and plate colony counting were adopted to explore the influences of different solvent pretreatments on the in vitro antibacterial efficacy of HH-0 and HH-50 supramolecular systems derived from the HQ-HL herb pair. Turbidimetric analysis revealed that the antibacterial activity of HH-50 was significantly higher than that of HH-0 at all tested concentrations (Fig. 1G). Further validation using the plate colony-counting method, as shown in the Fig. 1H, indicated that at a sample concentration of 0.55 mg·mL⁻^1^, no bacterial colonies were observed in the HH-50 group, whereas abundant colonies grew in the HH-0 group and Control group, which was consistent with the turbidimetric results. In summary, the lyophilized powder of HH-50 exhibited superior antibacterial activity to that of HH-0 at an equivalent mass.

Above results indicate that, compared with supramolecular assemblies obtained from the traditional water-based decoction system (HH-0), 50% ethanol-pretreated supramolecules (HH-50) exhibit distinct morphological changes at macroscopic and microscopic levels, as well as significantly enhanced antibacterial activity. These findings suggest that solvent pretreatment may modulate supramolecular characteristics, thereby altering their biological activities. Nevertheless, whether the antibacterial differences between HH-0 and HH-50 arise from variations in supramolecular characteristics or compositional discrepancies remains to be further elucidated.

### Chemical composition analysis of HH-0 and HH-50

**Fig. 2:**
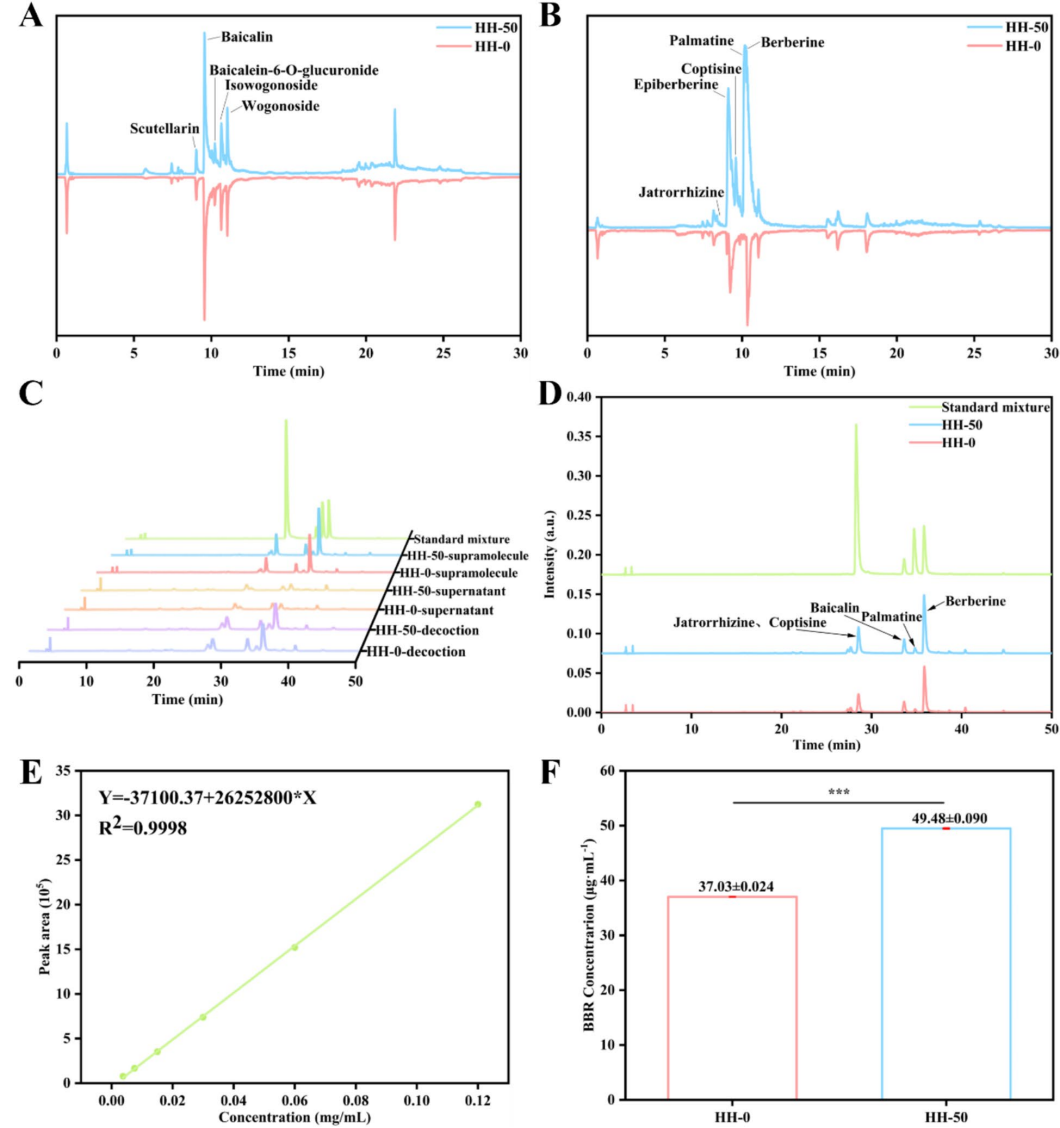
Chemical composition analysis of HH-0 and HH-50. **A** Total ion current chromatograms in the negative ion modes. **B** Total ion current chromatograms in the positive ion modes. **C** Liquid phase waterfall plot of HQ-HL water decoction and 50% ethanol-pretreated decoction with different fractions. **D** Liquid chromatograms of HH-0, HH-50 and the mixed standard. **E** Standard curve of berberine for content determination in samples. **F** Berberine content in HH-0 and HH-50 (n = 3)

To further clarify whether the discrepant antibacterial performance between HH-0 and HH-50 is related to their chemical compositional differences, HH-0 and HH-50 were qualitatively analyzed in the positive and negative ion modes by UHPLC-Q-Orbitrap HRMS, respectively. A total of 20 compounds were identified from the two samples (Fig. 2A, B), including 7 alkaloids, 11 flavonoids and 2 other compounds (Table 1) [10, 44]. And the results showed that HH-0 and HH-50 shared identical chemical component profiles.

**Table 1:** Identification of compounds in HH-0 and HH-50

No	Retention	Compound	Formula	Identity	Theoretical	Experimental	Mass accuracy	Fragment ion
1	1.05	Sucrose	C_12_H_22_O_11_	[M-H]^−^	341.1089	341.1068	−6.15	179.0549,119.0341,113.0236,89.0238,71.0133
2	7.43	Apigenin-6-C-arabinoside-8-C-glucoside	C_26_H_28_O_13_	[M-H]^−^	547.1458	547.1460	0.36	457.1144,427.1034,367.0822,337.0720
3	7.87	Chrysin-6-C-arabinoside-8-C-glucoside	C_26_H_28_O_13_	[M-H]^−^	547.1457	547.1460	0.55	457.1143,427.1035,367.0807,337.0703
4	9.17	Scutellarin	C_21_H_18_O_12_	[M-H]^−^	461.0725	461.0700	−5.42	285.0389,175.0238,113.0238
5	9.19	Verbascoside	C_29_H_36_O_15_	[M-H]^−^	623.1981	623.1946	−5.62	461.1639,161.0235,135.0444,113.0238
6	9.56	Baicalin	C_21_H_18_O_11_	[M-H]^−^	445.0774	445.0777	0.67	269.0452,253.1079,175.0238
7	10.23	Baicalein-6-O-glucuronide	C_21_H_18_O_10_	[M-H]^−^	445.0776	445.0777	0.22	269.0452,209.1177
8	10.65	Isowogonoside	C_22_H_20_O_11_	[M-H]^−^	459.0932	459.0935	0.65	283.0608,268.0371,175.0238
9	11.04	Wogonoside	C_22_H_20_O_11_	[M-H]^−^	459.0933	459.0933	0.00	283.0609,268.0371,175.0238
10	11.72	Baicalein	C_15_H_10_O_5_	[M-H]^−^	269.0455	269.0438	−6.32	239.0034,223.0385,213.0542,197.0594,171.0439,139.0026
11	11.92	5,6,7-Trihydroxy-8-methoxyflavone	C_16_H_12_O_6_	[M-H]^−^	299.0561	299.0548	−4.35	284.0312
12	12.5	Oroxylin A	C_16_H_12_O_5_	[M-H]^−^	283.0611	283.0598	−4.59	268.0361,239.0336,165.9897
13	0.92	Arginine	C_6_H_14_N_4_O_2_	[M + H]^+^	175.1189	175.1196	3.99	130.0980,116.0710,70.0653,60.0558
14	6.02	N-Nornuciferine	C_20_H_24_NO_4_^+^	[M]^+^	342.1705	342.1710	1.46	297.1129,282.0893,265.0867
15	7.96	Magnoflorine	C_20_H_24_NO_4_^+^	[M]^+^	342.1699	342.1719	5.84	297.1139,282.0904,265.0875,237.0924
16	8.68	Jatrorrhizine	C_20_H_20_NO_4_^+^	[M]^+^	338.1392	338.1396	1.18	322.1082,306.1138,278.1178
17	9.11	Epiberberine	C_20_H_18_NO_4_^+^	[M]^+^	336.1236	336.1238	0.59	320.0929,292.0989
18	9.37	Coptisine	C_19_H_14_NO_4_^+^	[M]^+^	320.0917	320.0927	3.12	292.0976,277.0739,262.0869
19	10.08	Palmatine	C_21_H_22_NO_4_^+^	[M]^+^	352.1543	352.1549	1.70	336.1240,322.1085,308.1290,294.1129
20	10.17	Berberine	C_20_H_18_NO_4_^+^	[M]^+^	336.1236	336.1241	1.49	321.0997

To further identify the key components responsible for the differences of supramolecular characteristics between HH-0 and HH-50, HPLC was employed to systematically analyze the main constituents across different decoction fraction, as well as to quantitatively determine the contents of representative antibacterial active ingredients. The results demonstrated that the chemical constituents found in the supernatants, supramolecules and whole decoction were identical in both water decoction and 50% ethanol-pretreated decoction (Fig. 2C), with jatrorrhizine (27.937 min), coptisine (28.038 min), baicalin (33.714 min), palmatine (34.787 min), and berberine (35.889 min) identified as the core components (Fig. 2D). Furthermore, the content of berberine, the key antibacterial active constituent in HH-0 and HH-50, were determined. As shown in Fig. 2E and F, the concentration in HH-0 was 37.03 ± 0.024 μg·mL⁻^1^, whereas the average content of berberine in the HH-50 was 49.48 ± 0.090 μg·mL⁻^1^. Accordingly, the berberine content in HH-50 was 1.35 times as high as that in HH-0.

These results demonstrated that HH-0 and HH-50 shared identical types of chemical constituents, yet noticeable discrepancies were observed in the content of berberine, the primary antibacterial active substance.

### Antibacterial activities of HH-0 and HH-50 with equivalent berberine content

**Fig. 3:**
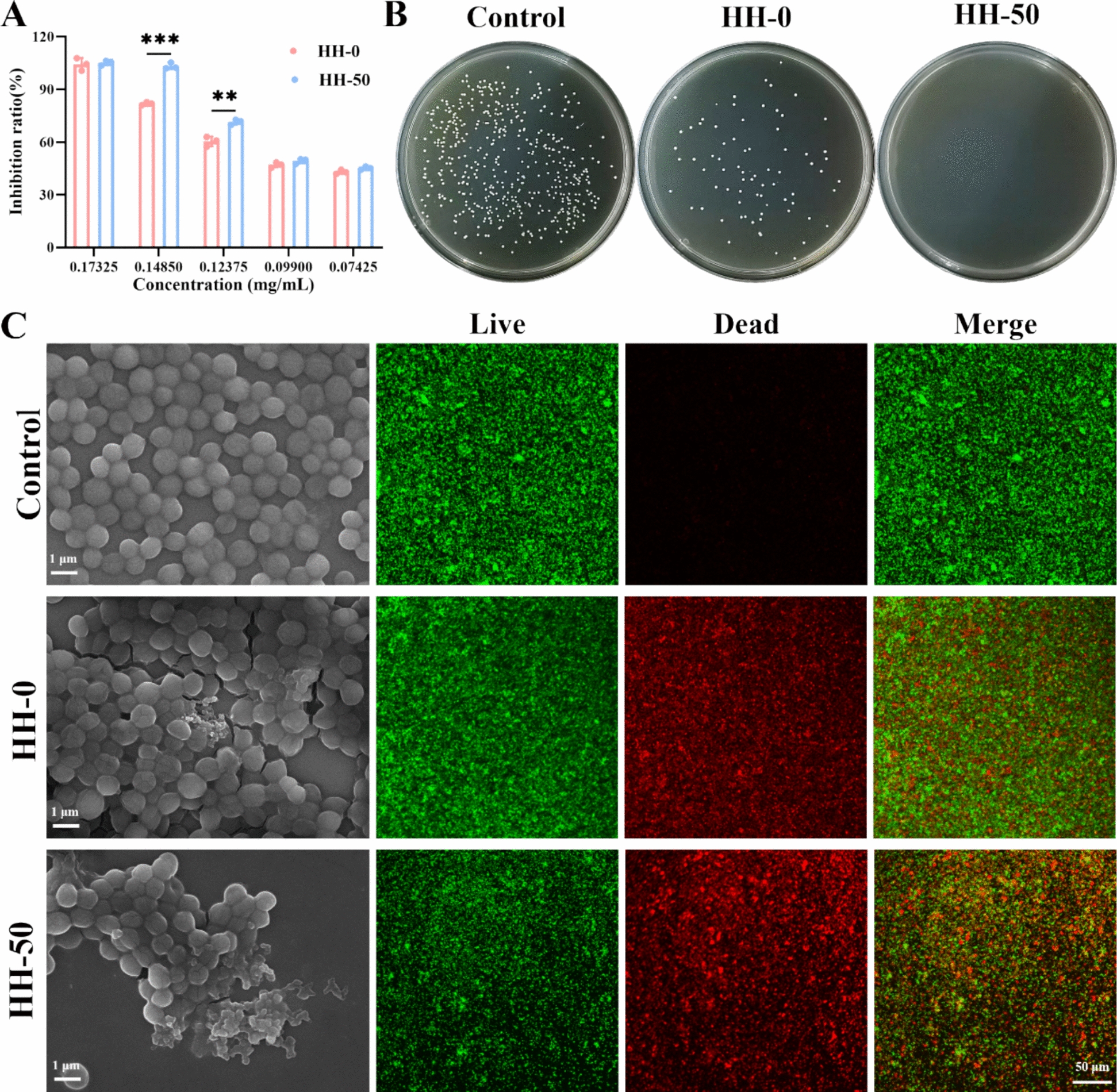
Anti-MRSA inhibitory effects of HH-50 and HH-0 with identical berberine contents. **A** The inhibition rate of MRSA at different concentrations (n = 3). **B** MRSA growth of different drug groups at 0.14850 mg·mL⁻^1^ (BBR). **C** SEM and live/dead bacterial staining images of MRSA treated with Control, HH-0 and HH-50

Considering the varied berberine levels and potential effects of different supramolecular assemblies on antibacterial activity, antibacterial assays at uniform berberine doses were further conducted to explore whether different supramolecular characteristics dominate the antibacterial differences between samples. The inhibitory effects of HH-0 and HH-50 on MRSA were determined by turbidimetry at equivalent berberine (BBR) contents, as shown in Fig. 3A. At a high BBR concentration of 0.17325 mg·mL⁻^1^, both supramolecules exhibited strong inhibitory activity against MRSA, with 100% inhibition rates and no significant difference between groups. At moderate BBR concentrations (0.14850 and 0.12375 mg·mL⁻^1^), the antibacterial activity of HH-50 was significantly superior to that of HH-0. At low BBR concentrations (0.09900 and 0.07425 mg·mL⁻^1^), both supramolecules showed weak inhibitory activity with no observable difference. Further validation using the plate colony-counting method (Fig. 3B) revealed that at a BBR concentration of 0.14850 mg·mL⁻^1^, no bacterial colonies were observed in the HH-50 group, whereas abundant colonies appeared in the HH-0 group and Control group, which was consistent with the turbidimetric results. The above results showed that HH-50 had better inhibitory effect on MRSA than HH-0. Morphological observation revealed that MRSA in the control group maintained intact cell morphology with a regular structure and smooth surface. In contrast, MRSA in both administration groups exhibited varying degrees of cellular damage, including cytoplasmic shrinkage, membrane disruption, and structural deformation. Notably, the bacterial cells in the HH-50 group showed more severe shrinkage and damage compared with those in the HH-0 group. Furthermore, it was observed that the supramolecules HH-50 had a higher degree of adhesion to MRSA cells than HH-0 (Fig. 3C). This enhanced bacterial adhesion capacity may promote the direct interaction between the supramolecular assembly and bacteria, thereby increasing the local concentration of active components around bacterial cells and strengthening the antibacterial effect, which is another key reason for the superior antibacterial activity of HH-50 over HH-0. Live/dead bacterial staining further confirmed that the antibacterial efficacy of HH-50 was superior to that of HH-0. Green fluorescence indicates live bacteria, while red fluorescence indicates dead bacteria. As shown in the Fig. 3C, obvious red fluorescence was observed in both the HH-50 and HH-0 groups, but the intensity of red fluorescence in the HH-50 group was significantly stronger than that in the HH-0 group. In contrast, distinct green fluorescence was observed in the Control group, indicating that most bacteria in the Control group remained viable, while both HH-0 and HH-50 could induce bacterial death, with HH-50 showing a stronger bactericidal effect.

Even at identical berberine concentrations, distinct differences in anti-MRSA efficacy still remained between HH-50 and HH-0. This phenomenon demonstrates that the antibacterial discrepancy between HH-50 and HH-0 is not merely governed by active ingredient contents, whereas variations in supramolecular assemblies exert an independent and notable regulatory effect on bacteriostatic activity.

### Differences in supramolecular assembly

**Fig. 4:**
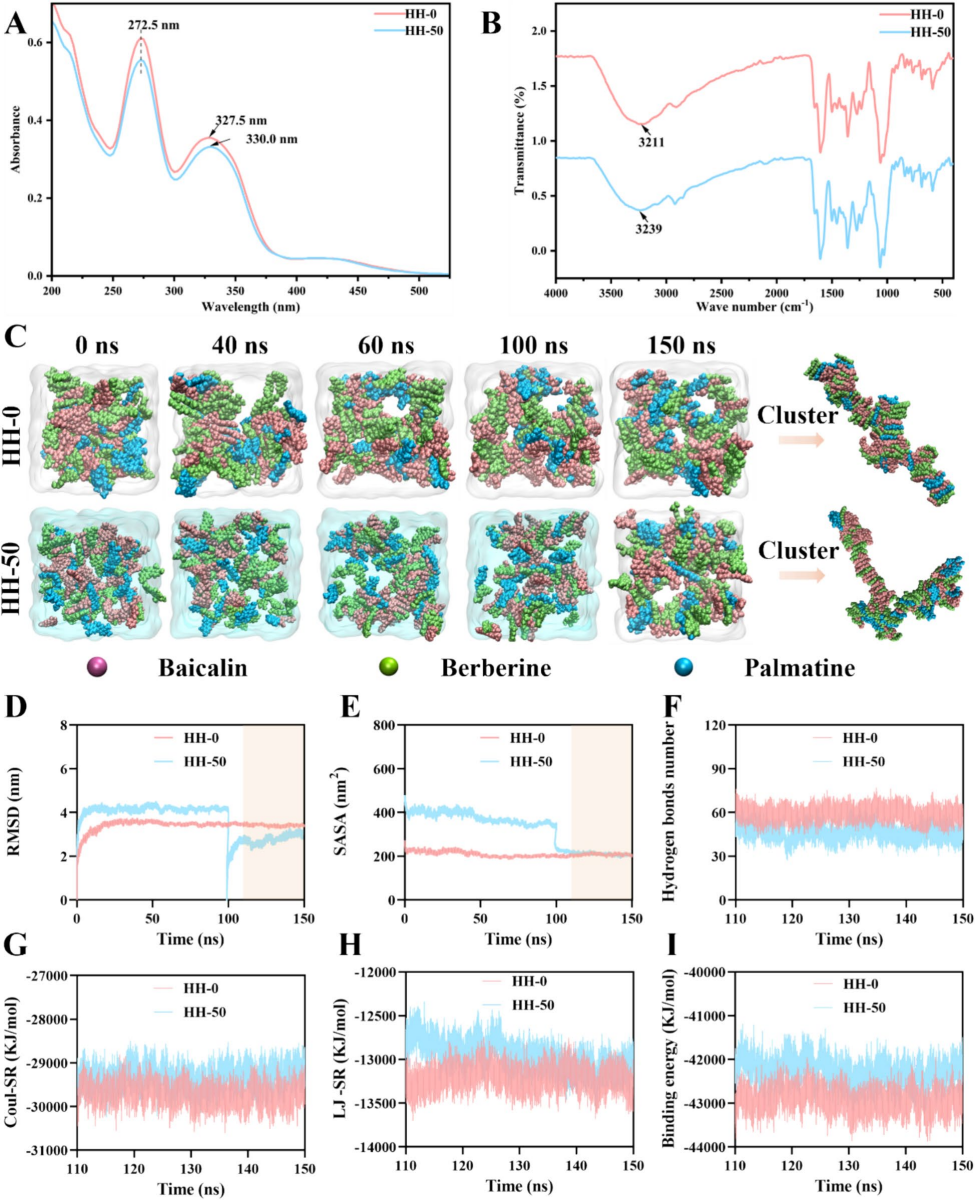
The assembly behavior analysis between HH-0 and HH-50. **A** UV spectroscopy. **B** IR spectroscopy. **C** MD simulation view of HH-0 and HH-50 at five different time point. **D** RMSD of HH-0 and HH-50. **E** SASA of HH-0 and HH-50. **F** Hydrogen bonds number of HH-0 and HH-50. **G** Coul-SR of HH-0 and HH-50. **H** LJ-SR of HH-0 and HH-50. **I** Binding energy of HH-0 and HH-50

To further unravel the underlying mechanism whereby differences in supramolecular assemblies result in divergent anti-MRSA efficacy, systematic supramolecular assembly analyses were performed. UV–visible absorption spectroscopy and Fourier-transform infrared spectroscopy are widely employed to characterize supramolecular interactions in traditional Chinese medicine. By analyzing variations in absorption peak position, intensity, and shape, the assembly behavior of supramolecular systems can be revealed. As shown in Fig. 4A, HH-0 and HH-50 shared an identical absorption peak at 272.5 nm, while the absorption peak of HH-50 at 330.0 nm showed a red shift relative to that of HH-0 at 327.5 nm, indicating a change in the energy gap of intermolecular π-π* transitions. Previous studies have demonstrated that the formation of HQ-HL supramolecular assemblies is closely associated with π-π stacking interactions between flavonoids and isoquinoline alkaloids [21]. Both HH-0 and HH-50 displayed hydroxyl absorption bands originating from flavonoid aglycones and glucuronic acid moieties. The hydroxyl peak of HH-50 was observed at 3239 cm⁻^1^ (Fig. 4B), showing a blue shift and decreased intensity compared with that of HH-0 (3211 cm⁻^1^), implying weakened intermolecular hydrogen-bonding interactions in HH-50 relative to HH-0. These results suggested that solvent pretreatment may alter intermolecular packing arrangements and modify the chemical microenvironment of functional groups, thereby giving rise to distinct supramolecular assembly.

To deeply elucidate the differences in assembly characteristics between the HH-0 and HH-50 supramolecular systems, molecular dynamics (MD) simulations were performed using 50 BA, 50 BBR, and 30 PA molecules to characterize their self-assembly behaviors. As illustrated in the Fig. 4C, after 150 ns MD simulation, the conformations of the two systems obtained by cluster analysis of the last 1 ns trajectory differed remarkably, in which HH-0 presented a linear structure and HH-50 displayed an L-shaped configuration. Moreover, as depicted in Fig. 4D and E, when the solvent molecules of the HH-50 system were switched from 50% ethanol to water at 100 ns, abrupt changes were observed in its RMSD and SASA values. In contrast, the RMSD and SASA values of HH-0 remained relatively stable throughout the simulation, as this system was always immersed in an aqueous environment. Both systems maintained steady RMSD and SASA values during the last 40 ns, indicating that equilibrium had been reached. Accordingly, the non-covalent interactions of the HH-0 and HH-50 systems were analyzed in detail during the final 40 ns. Specifically, HH-50 exhibited significantly fewer hydrogen bonds than HH-0, suggesting that HH-50 possessed more exposed functional groups prone to hydrogen bonding such as carboxyl groups (Fig. 4F). Meanwhile, HH-50 also displayed notably weaker binding energy (Fig. 4I), electrostatic interactions, and van der Waals forces relative to HH-0 (Fig. 4G, H). These results indicated that HH-50 possesses a relatively loose and flexible supramolecular architecture, whereas HH-0 exhibits a more compact and rigid self-assembled structure. Collectively, these distinct self-assembly characteristics indicate that HH-50 forms a more loose and dispersed supramolecular structure compared with HH-0, which may enable HH-50 to expose more accessible functional groups to bind with bacterial components, thereby facilitating its anti-MRSA efficacies.

### Metabolic and supramolecular regulatory mechanisms against MRSA

**Fig. 5:**
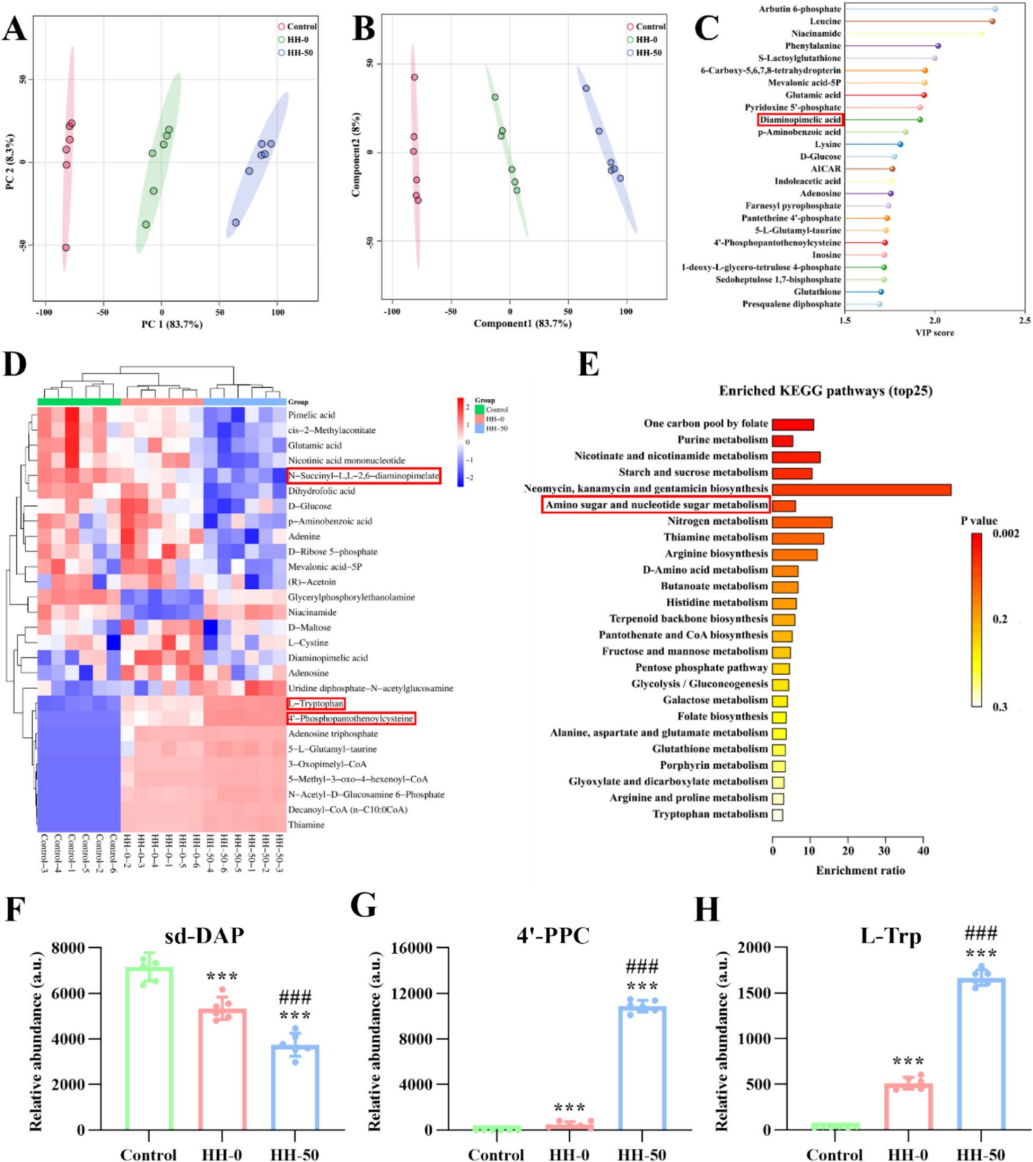
Non-targeted metabolomic analysis. **A** PCA of metabolites. **B** PLS-DA score plot. **C** Top 25 metabolites based on VIP score. **D** Hierarchical clustering results of 28 metabolites. **E** KEGG enrichment analysis. Abundance levels of metabolites SD-DAP (**F**), 4'-PPC (**G**) and L-Trp (**H**). ^***^p < 0.001, ^**^p < 0.01, ^*^p < 0.05 compared with Control group; ^###^*p* < 0.001, ^##^*p* < 0.01, ^#^*p* < 0.05 compared with HH-0 group

To clarify the potential antibacterial mechanisms linked to supramolecular assembly differences, untargeted metabolomics was adopted to explore how such supramolecular configuration variations drive divergent anti-MRSA effects of the *Scutellariae Radix*-*Coptidis Rhizoma* supramolecular system. Firstly, principal component analysis (PCA) (Fig. 5A) and partial least squares-discriminant analysis (PLS-DA) (Fig. 5B) showed that both HH-0 and HH-50 were significantly separated from the Control group, and in contrast to HH-0, the distribution region of HH-50 was more distant from that of the Control group. This phenomenon directly reflected a positive correlation between the degree of metabolic disturbance of MRSA by the supramolecules and their antibacterial efficacy. Permutation test showed that the intercept of the Q2 regression line was −0.238 (Fig. S1), which further verified the robustness of the model, excluded overfitting interference, and ensured the reliability of subsequent differential analysis results.

Collectively, comparisons of HH-50 vs Control and HH-50 vs HH-0 identified 28 differential metabolites (Table 2). Both VIP score plot and hierarchical clustering analysis of the heatmap and KEGG pathway enrichment analysis revealed that the differential metabolites were mainly enriched in pathways related to bacterial cell wall synthesis, energy metabolism, and amino acid metabolism (Fig. 5C, D and E). Diaminopimelic acid, a key precursor for bacterial cell wall peptidoglycan biosynthesis, exhibited prominent importance in the VIP score plot (Fig. 5C). Meanwhile, amino sugar and nucleotide sugar metabolism displayed the highest enrichment ratio, which directly sustains the synthesis of peptidoglycan sugar backbone and cell wall glycosylation homeostasis [45]. These lines of evidence suggested that the antibacterial effect of HH-50 was closely related to the pronounced disturbance of cell wall related metabolic pathways in MRSA.

**Table 2:** Databases of 28 potential differential metabolites

Compound	KEGG ID	HMDB	PubChem	HH-50 *vs* HH-0		
				log2FC	*P* value	Trend
4'-Phosphopantothenoylcysteine	C04352	HMDB0001117	440,304	4.5341	3.2257E-10	Up
L-Tryptophan	C00078	HMDB0000929	6305	1.7027	4.7762E-10	Up
5-L-Glutamyl-taurine	C05844	HMDB0004195	68,759	1.4411	4.7724E-04	Up
Thiamine	C00378	HMDB0000235	1130	1.2816	2.1252E-04	Up
5-Methyl-3-oxo-4-hexenoyl-CoA	C16471	HMDB0060399	9,549,331	1.2787	1.7461E-04	Up
N-Acetyl-D-Glucosamine 6-Phosphate	C00357	HMDB0001062	440,996	1.1153	5.3425E-04	Up
3-Oxopimelyl-CoA	C06715	HMDB0012158	441,147	1.0538	1.3901E-04	Up
Uridine diphosphate-N-acetylglucosamine	C00043	HMDB0000290	445,675	1.0497	3.2135E-02	Up
Decanoyl-CoA (n-C10:0CoA)	C05274	HMDB0006404	440,615	0.8756	1.7996E-07	Up
Adenosine triphosphate	C00002	HMDB0000538	5957	0.7163	1.8804E-02	Up
Niacinamide	C00153	HMDB0001406	936	0.6857	1.0699E-06	Up
Glycerylphosphorylethanolamine	C01233	HMDB0000114	123,874	0.6379	9.4931E-07	Up
cis-2-Methylaconitate	C04225	HMDB0006357	3,080,625	−0.1210	2.7607E-03	Down
D-Ribose 5-phosphate	C00117	HMDB0001548	440,101	−0.1218	1.4796E-03	Down
Mevalonic acid-5P	C01107	HMDB0001343	439,400	−0.1299	1.1064E-02	Down
Nicotinic acid mononucleotide	C01185	HMDB0001132	121,992	−0.1308	2.8296E-03	Down
D-Maltose	C00208	HMDB0000163	10,991,489	−0.1340	4.5001E-02	Down
Pimelic acid	C02656	HMDB0000857	385	−0.1827	4.2561E-03	Down
Adenine	C00147	HMDB0000034	190	−0.1871	3.8185E-03	Down
L-Cystine	C00491	HMDB0000192	67,678	−0.1873	4.1838E-02	Down
Dihydrofolic acid	C00415	HMDB0001056	98,792	−0.2280	2.9889E-04	Down
Glutamic acid	C00025	HMDB0000148	33,032	−0.2381	8.1999E-03	Down
D-Glucose	C00031	HMDB0000122	5793	−0.2845	1.7220E-02	Down
Adenosine	C00212	HMDB0000050	60,961	−0.3926	3.6554E-02	Down
Diaminopimelic acid	C00666	HMDB0001370	439,283	−0.4130	1.4992E-03	Down
(R)-Acetoin	C00810	HMDB0303161	439,314	−0.4763	2.4986E-02	Down
N-Succinyl-L,L-2,6-diaminopimelate	C04421	HMDB0012267	25,202,447	−0.5130	2.5819E-04	Down
p-Aminobenzoic acid	C00568	HMDB0001392	978	−0.8745	1.3128E-02	Down

From an in-depth analysis of the metabolic mechanism, the difference in antibacterial efficacy between the two treatment groups essentially stems from their distinct intensities of metabolic perturbation on key pathways of MRSA, leading to different degrees of bacterial damage. Heatmaps and bar charts of key metabolites showed that N-succinyl-L,L-2,6-diaminopimelic acid (SD-DAP), a key precursor in peptidoglycan synthesis for the MRSA cell wall [46], was significantly reduced in both treatment groups compared with the control, with the lowest level observed in the HH-50 group (Fig. 5F). This indicates that HH-50, the formulation with higher efficacy, exerts a stronger inhibitory effect on bacterial peptidoglycan biosynthesis, thereby more effectively disrupting cell wall integrity and ultimately causing bacterial lysis and death. Notably, the metabolic process of SD-DAP is closely associated with amino sugar and nucleotide sugar metabolism, which further confirms that HH-50 exerts its antibacterial effect mainly via interfering with peptidoglycan metabolic pathways.

In addition, 4'-phosphopantetheine (4'-PPC) and L-tryptophan (L-Trp) were significantly upregulated in both treatment groups, with higher relative abundances in HH-50 than in HH-0 (Fig. 5G, H). These two metabolites are key intermediates in pantothenate/CoA biosynthesis and tryptophan metabolism, respectively. Their abnormal accumulation suggests that the formulations block coenzyme synthesis and amino acid metabolism in MRSA. Notably, HH-50 inhibits these two pathways more comprehensively, effectively interrupting bacterial energy supply (as CoA acts as a central coenzyme in energy metabolism) and protein synthesis (as tryptophan is an essential amino acid for bacterial proteins). This constitutes another crucial mechanism underlying the enhanced anti-MRSA efficacy of HH-50.

**Fig. 6:**
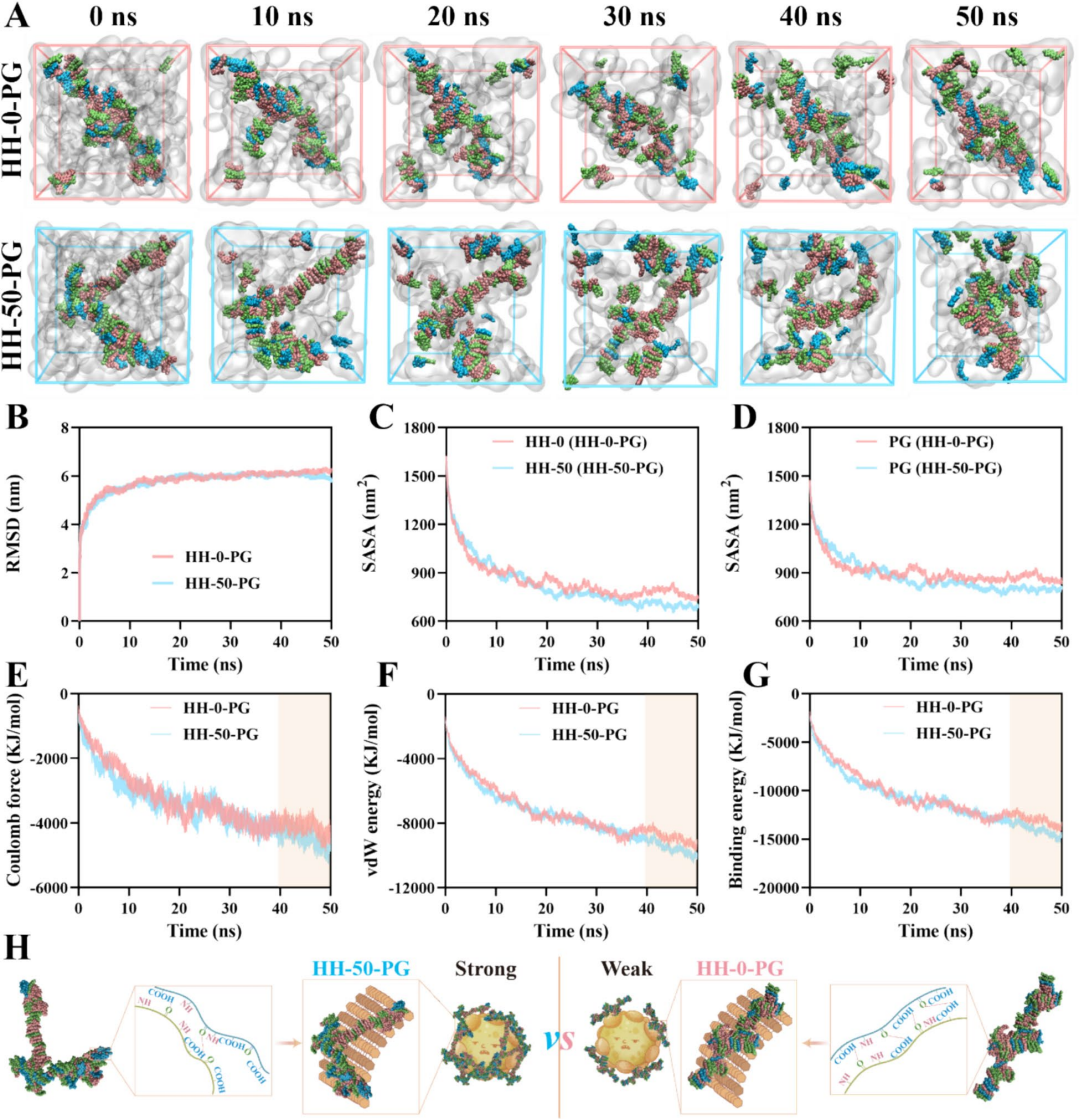
MD simulations of HH-0 and HH-50 binding to bacterial peptidoglycan. **A** MD simulations views of the interactions between the supramolecular assemblies and peptidoglycan at six different time points. **B** RMSD analysis. **C** Time-dependent SASA of HH-0 and HH-50 alone upon interaction with peptidoglycan. **D** Time-dependent SASA of peptidoglycan upon interaction with HH-0 and HH-50, respectively. **E** Coul-SR analysis. **F** LJ-SR analysis. **G** Binding energy analysis. **H** Schematic diagram of antibacterial differences between HH-0 and HH-50 caused by distinct supramolecular assembly differences

Based on the aforementioned results, it was observed that HH-50 exerted stronger regulatory effects on metabolic pathways associated with MRSA peptidoglycan biosynthesis. Therefore, to further clarify the structural basis underlying the differences in antibacterial efficacy between the two supramolecular systems, this study systematically analyzed the interaction characteristics between HH-0, HH-50 and peptidoglycan (PG), which is the key component of the MRSA cell wall, by means of molecular dynamics (MD) simulations (Fig. 6A). RMSD (Fig. 6B) and SASA (Fig. S2) parameters of the HH-0-PG and HH-50-PG systems gradually converged and maintained stable within the final 10 ns, confirming that the simulation systems achieved equilibrium. Subsequent SASA analysis on individual supramolecules and peptidoglycan demonstrated that both SASA values of HH-50 and peptidoglycan were significantly reduced after intermolecular binding. During the equilibrated 10 ns interval, they remained lower than those of the HH-0 and peptidoglycan in HH-0-PG system respectively, reflecting tighter adhesion and binding between HH-50 and peptidoglycan (Fig. 6C, D). Further calculation of binding energy over the stable 10 ns trajectory showed that HH-50-PG exhibited higher binding affinity, with remarkably enhanced electrostatic and van der Waals interactions relative to HH-0-PG (Fig. 6E, F and G). Such stronger intermolecular interactions facilitate higher local drug aggregation around MRSA cells, thereby improving the antibacterial activity of HH-50. The results are highly consistent with the structural features of HH-50, including fewer intramolecular hydrogen bonds and more exposed carboxyl groups, which facilitate more extensive intermolecular interactions with peptidoglycan. As Fig. 6H illustrated, the divergent antibacterial effects between HH-0 and HH-50 stem from their distinct assembly differences. Overall, the loose assembly and abundant exposed functional groups of HH-50 strengthen its affinity toward bacterial cell walls, promote local enrichment of active ingredients such as berberine, and ultimately account for its enhanced antibacterial efficacy.

These findings suggest that the differential antibacterial efficacy between the two supramolecular systems was tightly associated with their distinct supramolecular assembly patterns and interfacial interactions toward bacterial cell walls. This clearly demonstrates the typical phenomenon of chemical equivalence yet biological nonequivalence.

## Discussion

The present study demonstrated that beyond the conventional chemical perspective, different solvent pretreatments (water and 50% ethanol) significantly regulate the supramolecular self-assembly behavior of the *Scutellariae Radix*-*Coptidis Rhizoma* herbal pair, alter the interaction modes between supramolecular aggregates and MRSA, and ultimately mediate the differential anti-MRSA efficacy. Distinct from most previous studies that merely focused on the categories and contents of active components obtained via different extraction methods, the core innovation of the current study is the confirmation that the pharmacological activity of herbal medicines is not solely determined by chemical composition; supramolecular structure also serves as a core determinant dominating efficacy difference. Consistent with the consensus of existing comparative studies on water and ethanol extracts of this herbal pair, ethanolic extraction preferentially enriches lipophilic aglycones and free alkaloid monomers, while aqueous extraction tends to retain polar flavonoid components, thereby forming obviously differentiated chemical profiles [25–29]. Nevertheless, previous studies have long been limited to qualitative and quantitative analysis of small-molecule components, while neglecting the crucial effects of extraction solvents on supramolecular assembly and structural characteristics, which fails to fully elaborate the material basis underlying efficacy discrepancies among different extraction technologies.

Accumulating supramolecular studies have verified that solvent exerts irreversible regulatory effects on the self-assembly properties of ingredients. Even when finally reconstituted in the same aqueous system, the solvent-induced molecular binding patterns and aggregation characteristics cannot be completely eliminated [47, 48]. This phenomenon originates from the thermodynamic modulation of intermolecular forces by solvents. The polarity, dielectric constant, and solvation capacity of different solvents systematically alter the intensity of intermolecular hydrogen bonds, electrostatic interactions, hydrophobic forces, and π-π stacking, thereby regulating molecular dissociation, diffusion, and recombination behaviors and ultimately determining the nucleation and growth of supramolecular aggregates [6–8, 32]. Such differences in assembly pathways lead to significant variations in the microscopic morphology, particle size, structural stability, and interfacial properties of aggregates. These structural discrepancies further affect the solubility, structural stability, bioavailability, and target-binding capacity of supramolecular assemblies, consequently altering their pharmacological performance [2, 38, 43].

In this study, a multi-dimensional framework combining material characterization and mechanistic analysis was established to systematically elaborate the solvent-mediated supramolecular differentiation and corresponding anti-MRSA mechanisms between water-pretreated (HH-0) and 50% ethanol-pretreated (HH-50) supramolecules. Macroscopic and microscopic morphological analyses revealed that HH-50 aggregates exhibited a rougher surface and higher zeta potential than HH-0, directly demonstrating that pretreatment solvents reshape the microscopic supramolecular morphology. UHPLC-Q-Orbitrap HRMS and HPLC analyses further confirmed that HH-0 and HH-50 shared identical component categories, while the berberine content in HH-50 was 1.35-fold higher than that in HH-0. Notably, antibacterial assays performed with identical berberine concentrations definitively verified that the differential anti-MRSA activities between HH-0 and HH-50 originated from their intrinsic supramolecular structural differences, rather than component concentration disparities. UV–vis and FT-IR spectroscopy combined with molecular dynamics simulations confirmed that solvent pretreatment substantially modulates intermolecular non-covalent interactions and the thermodynamic stability of self-assembled aggregates. Furthermore, untargeted metabolomics and supramolecular-peptidoglycan interaction simulations revealed that structurally distinct supramolecular assemblies possess varied binding affinities toward MRSA peptidoglycan, thereby inducing differential patterns of cell wall destruction and metabolic homeostasis disturbance. These findings systematically clarify the chained regulatory mechanism of “solvent pretreatment–supramolecular structure-antibacterial efficacy”.

Several limitations of this study should be acknowledged. First, only two pretreatment systems (pure water and 50% ethanol) were investigated in the present work. It remains unclear whether gradient ethanol concentrations can continuously modulate the dissociation, aggregation, morphological features, and dynamic assembly behaviors of *Scutellariae Radix*-*Coptidis Rhizoma* supramolecules. Second, the current study only focused on the anti-MRSA efficacy derived from solvent-induced supramolecular variations. Whether other pharmacological activities of this herbal pair, such as anti-inflammatory and antioxidant effects, are modulated by supramolecular configuration requires further experimental validation.

In summary, this study breaks through the conventional research paradigm that merely emphasizes component categories and contents when comparing different herbal extraction methods. Our findings clearly demonstrate that pretreatment solvents exert irreversible modulation on supramolecular self-assembly and ultimately alter the anti-MRSA performance of *Scutellariae Radix*-*Coptidis Rhizoma*, even after uniform reconstitution in an aqueous environment. This study reveals the essential differences in the pharmacological material basis between traditional water decoction and modern ethanol-containing extraction from the perspective of solvent-regulated supramolecular assembly, providing a novel theoretical insight for interpreting the material basis of herbal efficacy and optimizing modern extraction technologies of traditional Chinese medicine.

## Conclusion

In conclusion, our findings reveal that solvent pretreatment serves as a pivotal regulatory factor governing supramolecular assembly and biological activities of the *Scutellariae Radix*-*Coptidis Rhizoma* herb pair. While UHPLC-Q-Orbitrap HRMS chemical profiling verified consistent chemical constituents between the water and 50% ethanol-pretreated systems, remarkable disparities existed in their supramolecular assembly features. Specifically, compared with compact smooth-surfaced aggregates in the aqueous HH-0 system, 50% ethanol-pretreated supramolecules (HH-50) presented distinctive architectures decorated with abundant surface-protruding nanoparticles. Crucially, the preferable anti-MRSA performance of HH-50 relative to HH-0 under equivalent berberine concentrations corroborates that the biological potency of herbal supramolecules is inherently dependent on their self-assembly patterns. These results indicate that the divergent anti-MRSA activities of the two supramolecular assemblies are closely correlated with their distinctive self-assembly behaviors as well as interfacial binding interactions toward bacterial peptidoglycan, which well embodies the phenomenon of chemical equivalence accompanied by biological nonequivalence. This work highlights that extraction solvents function not merely as dissolution media, but also as structural templates that modulate interfacial interaction modes of herbal supramolecular complexes. The present findings further enrich the theoretical interpretation of TCM chemical connotations, and provide robust scientific evidence for the optimized development and precise formulation design of clinical herbal preparations.

## Supplementary Information

Supplementary Material 1.

## Data Availability

No datasets were generated or analysed during the current study.

## Abbreviations

HQ: *Scutellariae* *Radix*
HL: *Coptidis* *Rhizoma*
HH-0: Water-extracted supramolecules of HQ and HL
HH-50: 50% Ethanol-pretreated supramolecules of HQ and HL
MRSA: Methicillin-resistant *Staphylococcus* *aureu*s
UHPLC-Q-Orbitrap HRMS: Ultra-high performance liquid chromatography-Q Exactive quadrupole-Orbitrap high-resolution mass spectrometry
HPLC: High-performance liquid chromatography
SEM: Scanning electron microscope

## References

[1] Gao Y, Dong YY, Guo Q, Wang HH, Feng M, Yan Z, et al. Study on supramolecules in traditional Chinese medicine decoction. Molecules. 2022;27:3268. 35630743 10.3390/molecules27103268 PMC9144598

[2] Hou Y, Zou LJ, Li QL, Chen MY, Ruan HN, Sun ZC, et al. Supramolecular assemblies based on natural small molecules: union would be effective. Mater Today Bio. 2022;15:100327. 35757027 10.1016/j.mtbio.2022.100327 PMC9214787

[3] Lin XY, Huang XM, Tian XH, Yuan ZH, Lu JH, Nie XQ, et al. Natural small-molecule-based carrier-free self-assembly library originated from traditional Chinese herbal medicine. ACS Omega. 2022;7:43510–21. 36506183 10.1021/acsomega.2c04098 PMC9730315

[4] Hu J, Wu ZS, Yan JJ, Pang WS, Liang DH, Xu XJ. A promising approach for understanding the mechanism of Traditional Chinese Medicine by the aggregation morphology. J Ethnopharmacol. 2009;123:267–74. 19429371 10.1016/j.jep.2009.03.007

[5] Wan SY, Gao YT, Zhang ZB, Wu FY, Zheng ZY, Chen HS, et al. Oriented linear self-assembly of colloidal nanocrystals through regioselective formation of hydrogen-bonded supramolecular bridges. J Am Chem Soc. 2024;146:14225–34. 38717289 10.1021/jacs.4c03457

[6] Xie WH, Ren Y, Jiang FL, Huang XY, Yu BJ, Liu JH, et al. Solvent-pair surfactants enabled assembly of clusters and copolymers towards programmed mesoporous metal oxides. Nat Commun. 2023;14:7913. 38129402 10.1038/s41467-023-44193-z PMC10739937

[7] Yang HK. Structure- and solvent-triggered influences in the self-assembly of polyoxometalate-steroid conjugates. RSC Adv. 2016;6:66431–7.

[8] Chakravarthy RD, Sahroni I, Wang CW, Mohammed M, Lin HC. Temperature-induced nanostructure transition for supramolecular gelation in water. ACS Nano. 2023;17:11805–16. 37294326 10.1021/acsnano.3c02753

[9] Huang XM, Liu XJ, Lin XY, Yuan ZH, Zhang YZ, Wang ZJ, et al. Thermodynamics driving phytochemical self-assembly morphological change and efficacy enhancement originated from single and co-decoction of traditional Chinese medicine. J Nanobiotechnol. 2022;20:322. 10.1186/s12951-022-01734-w PMC9743513 36510210

[10] Wei JC, Lin XY, Zhao YH, Tan XR, Wang ZX, Li YY, et al. Thermodynamic-driven supramolecular transition from nanofibers to nanospheres: morphology-dependent antibacterial specificity of herb medicines. Chin Med. 2025;20:147. 40993776 10.1186/s13020-025-01185-z PMC12462035

[11] Barravecchia L, Blanco-Gómez A, Neira I, Skackauskaite R, Vila A, Rey-Rico A, et al. “Vermellogens” and the development of CB[8]-based supramolecular switches using pH-responsive and non-toxic viologen analogues. J Am Chem Soc. 2022;144:19127–36. 36206443 10.1021/jacs.2c08575 PMC9682480

[12] Li DW, Tang GK, Yao H, Zhu YQ, Shi CG, Fu Q, et al. Formulation of pH-responsive PEGylated nanoparticles with high drug loading capacity and programmable drug release for enhanced antibacterial activity. Bioact Mater. 2022;16:47–56. 35386319 10.1016/j.bioactmat.2022.02.018 PMC8958631

[13] Lan JS, Liu L, Zeng RF, Qin YH, Hou JW, Xie SS, et al. Tumor-specific carrier-free nanodrugs with GSH depletion and enhanced ROS generation for endogenous synergistic anti-tumor by a chemotherapy-photodynamic therapy. Chem Eng J. 2021;407:127212.

[14] Su YG, Chen R, Wang BJ, Wang T, Tao JJ, Diao QJ, et al. Erythrocyte membrane camouflaged celastrol and bilirubin self-assembly for rheumatoid arthritis immunotherapy based on STING inhibition and RONS clearance. J Nanobiotechnol. 2025;23:3389. 10.1186/s12951-025-03389-9 PMC12032812 40287703

[15] Chen M, Wang PL, Li T, Li LS, Li JF, Bai H, et al. Comprehensive analysis of Huanglian Jiedu decoction: revealing the presence of a self-assembled phytochemical complex in its naturally-occurring precipitate. J Pharm Biomed Anal. 2021;195:113820. 33303266 10.1016/j.jpba.2020.113820

[16] Lin D, Du Q, Wang HQ, Gao GZ, Zhou JW, Ke LJ, et al. Antidiabetic micro-/nanoaggregates from Ge-Gen-Qin-Lian-Tang decoction increase absorption of Baicalin and cellular antioxidant activity in vitro. Biomed Res Int. 2017;2017:9217912. 28798936 10.1155/2017/9217912 PMC5536148

[17] Luo YT, Fu S, Liu YL, Kong SS, Liao Q, Li LF, et al. Banxia Xiexin decoction modulates gut microbiota and gut microbiota metabolism to alleviate DSS-induced ulcerative colitis. J Ethnopharmacol. 2024;326:117990. 38423412 10.1016/j.jep.2024.117990

[18] Xiao SW, Liu C, Chen MJ, Zou JF, Zhang ZM, Cui X, et al. *Scutellariae radix* and *coptidis rhizoma* ameliorate glycolipid metabolism of type 2 diabetic rats by modulating gut microbiota and its metabolites. Appl Microbiol Biotechnol. 2020;104:303–17. 31758238 10.1007/s00253-019-10174-w

[19] Huang WQ, Wang YX, Tian WS, Cui XX, Tu PF, Li J, et al. Biosynthesis investigations of terpenoid, alkaloid, and flavonoid antimicrobial agents derived from medicinal plants. Antibiotics. 2022;11:1380. 36290037 10.3390/antibiotics11101380 PMC9598646

[20] Millar BC, Rao JR, Moore JE. Fighting antimicrobial resistance (AMR): Chinese herbal medicine as a source of novel antimicrobials-an update. Lett Appl Microbiol. 2021;73:400–7. 34219247 10.1111/lam.13534

[21] Li T, Wang PL, Guo WB, Huang XM, Tian XH, Wu GR, et al. Natural berberine-based Chinese herb medicine assembled nanostructures with modified antibacterial application. ACS Nano. 2019;13:6770–81. 31135129 10.1021/acsnano.9b01346

[22] Lu YJ, Joerger R, Wu CQ. Study of the chemical composition and antimicrobial activities of ethanolic extracts from roots of Georgi. J Agr Food Chem. 2011;59:10934–42. 21866919 10.1021/jf202741x

[23] Meng X, Ning C, Kang MN, Wang XW, Yu ZY, Hao XY, et al. Baicalin: natural sources, extraction techniques, and therapeutic applications against bacterial infections. Molecules. 2025;30:3464. 40941991 10.3390/molecules30173464 PMC12429897

[24] Chinese Pharmacopoeia Commission. 2025. Pharmacopoeia of the People's Republic of China.

[25] Tong RS, Li JQ, Peng C, Zou J, Zhang K, Zhong CS. Study on water extracting and alcohol precipitation in banxia xiexin decoction for purging stomach-fire using orthogonal design. Liaoning J Tradit Chinese Med. 2012;39(1):70-72. (in Chinese)

[26] Liu S, Tang LQ, Chen LM, Li C, Zhang ST, Wang QM, et al. Study on extraction technology of berberine from rhizoma coptidis by the method of orthogonal-test optimization. China Pharm. 2004,15(1):18-19.(in Chinese)

[27] Zhong JB, Wang LL, Wang YY, Xie RH, Yin CC. Study on optimization of the extraction techniques of the total flavonoids from scutellariae baicalensis using response surface methodology. J Jiujiang University. 2014;69–74. (in Chinese)

[28] Wang SB, Zhang YC. In vitro inhibition of five Chinese herbal extracts and their combinations against Aeromonas hydrophila. Feed Research. 2023;80–85. (in Chinese)

[29] Chen ZZ, Dong CC, Wang YB. Extraction Methods and Related Antibacterial Performances of Effective Components from Rheum Palmatm, Phellodendron Amurense, Rhizoma Coptidis and Scutellaria Baicalensia. Flavour Fragrance Cosmetics. 2025;78–81. (in Chinese)

[30] Ma HT, Cheng XX, Zhang G, Miao TF, He ZX, Zhang W. Revealing pathway complexity and helical inversion in supramolecular assemblies through solvent-induced radical disparities. Adv Sci. 2024;11:2308371. 10.1002/advs.202308371 PMC11005740 38311583

[31] Maeda T, Okamura N, Das S, Ishida T, Kotera H, Ajayaghosh A, et al. Solvent-controlled supramolecular polymerization and morphology-depended photoconductivity modulation in a squaraine-naphthalene diimide-squaraine bulk p/n heterojunction. Angew Chem Int Ed. 2026;65:e16556. 10.1002/anie.202516556 41195966

[32] Zhang HL, Chan MHY, Lam J, Leung MY, Wu LX, Yam VWW. Solvation and temperature-modulated supramolecular assembly of amphiphilic water-soluble Schiff base-containing platinum(II) complexes. Org Chem Front. 2025;12:1544.

[33] Li SX, Yan XS, Zhang JQ, Guo X, Zhang YK, Su MH, et al. J-aggregation-driven supramolecular assembly of dye-conjugated block polymers: from morphological tailoring to anticancer applications. Adv Funct Mater. 2021;31:2105189.

[34] Lin RF, Li GY, He QF, Song JF, Ma YM, Zhan YT, et al. Synthesis of mesoporous catechin nanoparticles as biocompatible drug-free antibacterial mesoformulation. J Am Chem Soc. 2024;146:26983–93. 39294849 10.1021/jacs.4c08336

[35] Tian XX, Liu KJ, Wang PY. Flower-shaped supramolecular polymer enabling biofilm eradication, improved foliar affinity, and bacterial disease management. Angew Chem Int Ed. 2026;65:e25538. 10.1002/anie.202525538 41757571

[36] Brandenburg JG, Bannwarth C, Hansen A, Grimme S. B97-3c: a revised low-cost variant of the B97-D density functional method. J Chem Phys. 2018;148:064104. 29448802 10.1063/1.5012601

[37] Grimme S, Antony J, Ehrlich S, Krieg H. A consistent and accurate ab initio parametrization of density functional dispersion correction (DFT-D) for the 94 elements H-Pu. J Chem Phys. 2010;132:154104. 20423165 10.1063/1.3382344

[38] Weigend F, Ahlrichs R. Balanced basis sets of split valence, triple zeta valence and quadruple zeta valence quality for H to Rn: design and assessment of accuracy. Phys Chem Chem Phys. 2005;7:3297–305. 16240044 10.1039/b508541a

[39] Zhao Y, Lynch BJ, Truhlar DG. Doubly hybrid meta DFT: new multi-coefficient correlation and density functional methods for thermochemistry and thermochemical kinetics. J Phys Chem A. 2004;108:4786–91.

[40] Kossmann S, Neese F. Efficient structure optimization with second-order many-body perturbation theory: the RIJCOSX-MP2 method. J Chem Theory Comput. 2010;6:2325–38. 26613489 10.1021/ct100199k

[41] Wang JM, Wolf RM, Caldwell JW, Kollman PA, Case DA. Development and testing of a general amber force field. J Comput Chem. 2004;25:1157–74. 15116359 10.1002/jcc.20035

[42] Hsin J, Arkhipov A, Yin Y, Stone JE, Schulten K. Using VMD: an introductory tutorial. Curr Protoc Bioinf. 2008;24:5.7.1-5.7.48. 10.1002/0471250953.bi0507s24 PMC2972669 19085979

[43] Su YL, Ma LY, Wen Y, Wang H, Zhang SW. Studies of the antibacterial activities of several polyphenols against clinical isolates of methicillin-resistant. Molecules. 2014;19:12630–9. 25153875 10.3390/molecules190812630 PMC6271679

[44] Wang ZJ, Li W, Lu JH, Yuan ZH, Pi WM, Zhang YZ, et al. Revealing the active ingredients of the traditional Chinese medicine decoction by the supramolecular strategies and multitechnologies. J Ethnopharmacol. 2023;300:115704. 36096345 10.1016/j.jep.2022.115704

[45] Bouhss A, Trunkfield AE, Bugg TDH, Mengin-Lecreulx D. The biosynthesis of peptidoglycan lipid-linked intermediates. FEMS Microbiol Rev. 2008;32:208–33. 18081839 10.1111/j.1574-6976.2007.00089.x

[46] Campbell C, Fingleton C, Zeden MS, Bueno E, Gallagher LA, Shinde D, et al. Accumulation of succinyl coenzyme a perturbs the methicillin-resistant (MRSA) succinylome and is associated with increased susceptibility to beta-lactam antibiotics. MBio. 2021;12:e00864-e921. 10.1128/mBio.00530-21 PMC8437408 34182779

[47] Borsdorf L, Herkert L, Bäumer N, Rubert L, Soberats B, Korevaar PA, et al. Pathway-controlled aqueous supramolecular polymerization via solvent-dependent chain conformation effects. J Am Chem Soc. 2023;145:8882–95. 37053499 10.1021/jacs.2c12442

[48] van der Tol JJB, Vantomme G, Meijer EW. Solvent-induced pathway complexity of supramolecular polymerization unveiled using the Hansen solubility parameters. J Am Chem Soc. 2023;145:17987–94. 37530219 10.1021/jacs.3c05547 PMC10436269

