# Supplementary material for: Beyond chemical composition: solvent pretreatment modulates supramolecular assembly and interactions with MRSA to enhance antibacterial efficacy of Scutellariae Radix-Coptidis Rhizoma
Source: Chin Med. 2026 Jul 17;21:196. doi: 10.1186/s13020-026-01460-7 (PMC13378121; doi:10.1186/s13020-026-01460-7)
Supplement: Supplementary file 1 — Supplementary Material 1. [file 13020_2026_1460_MOESM1_ESM.docx]

**Supplementary material**


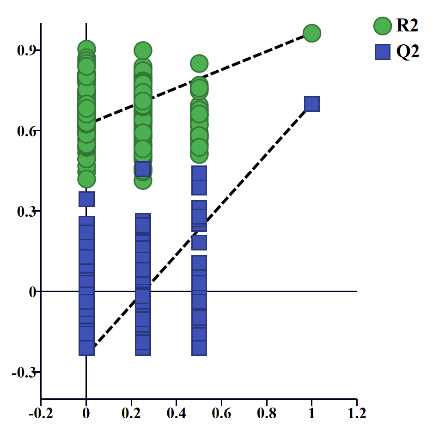


Fig. S1. Permutation tests of the PLS-DA models.


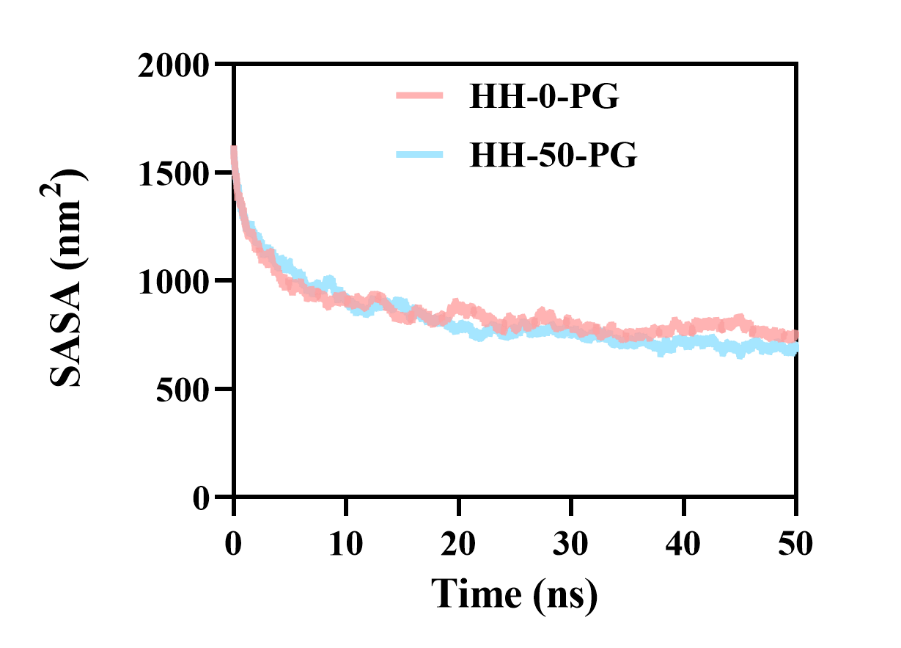


Fig. S2. Time-dependent total SASA of HH-0 and Assembly B in complex with peptidoglycan.
